# Supplementary material for: Genome-Wide Association Analysis of Freezing Tolerance and Winter Hardiness in Winter Wheat of Nordic Origin
Source: Plants (Basel). 2023 Nov 29;12(23):4014. doi: 10.3390/plants12234014 (PMC10708462; doi:10.3390/plants12234014)
Supplement: Supplementary file 1 [file plants-12-04014-s001.zip › GWAS_freezing_tolerance_wheat_supplementary_material_v3.pdf]

plants

MDPI

Supplementary material

Genome-Wide Association Analysis of Freezing Tolerance and Winter Hardiness in Winter Wheat of Nordic Origin

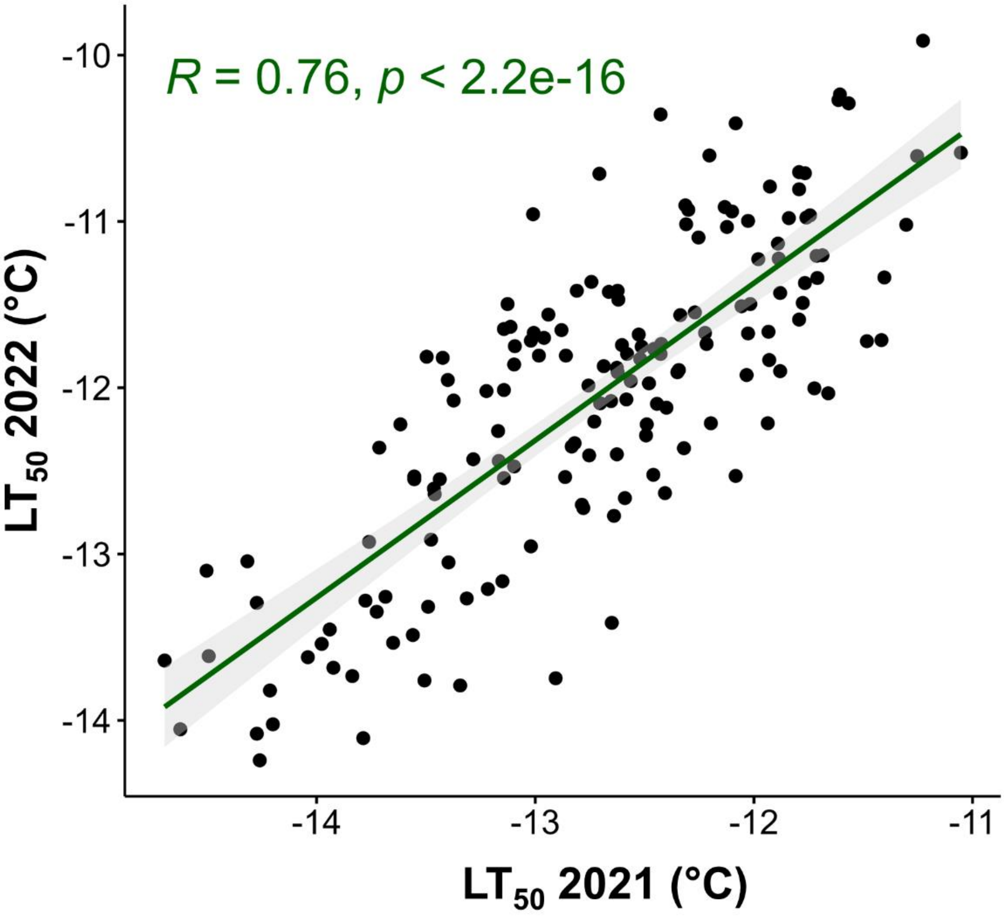

Figure S1. Relationship between LT_{50} (temperature where 50% of plants are killed) values under controlled conditions of 160 winter wheat cultivars and landraces of Nordic origin, obtained from experiments carried out in 2021 and 2022.

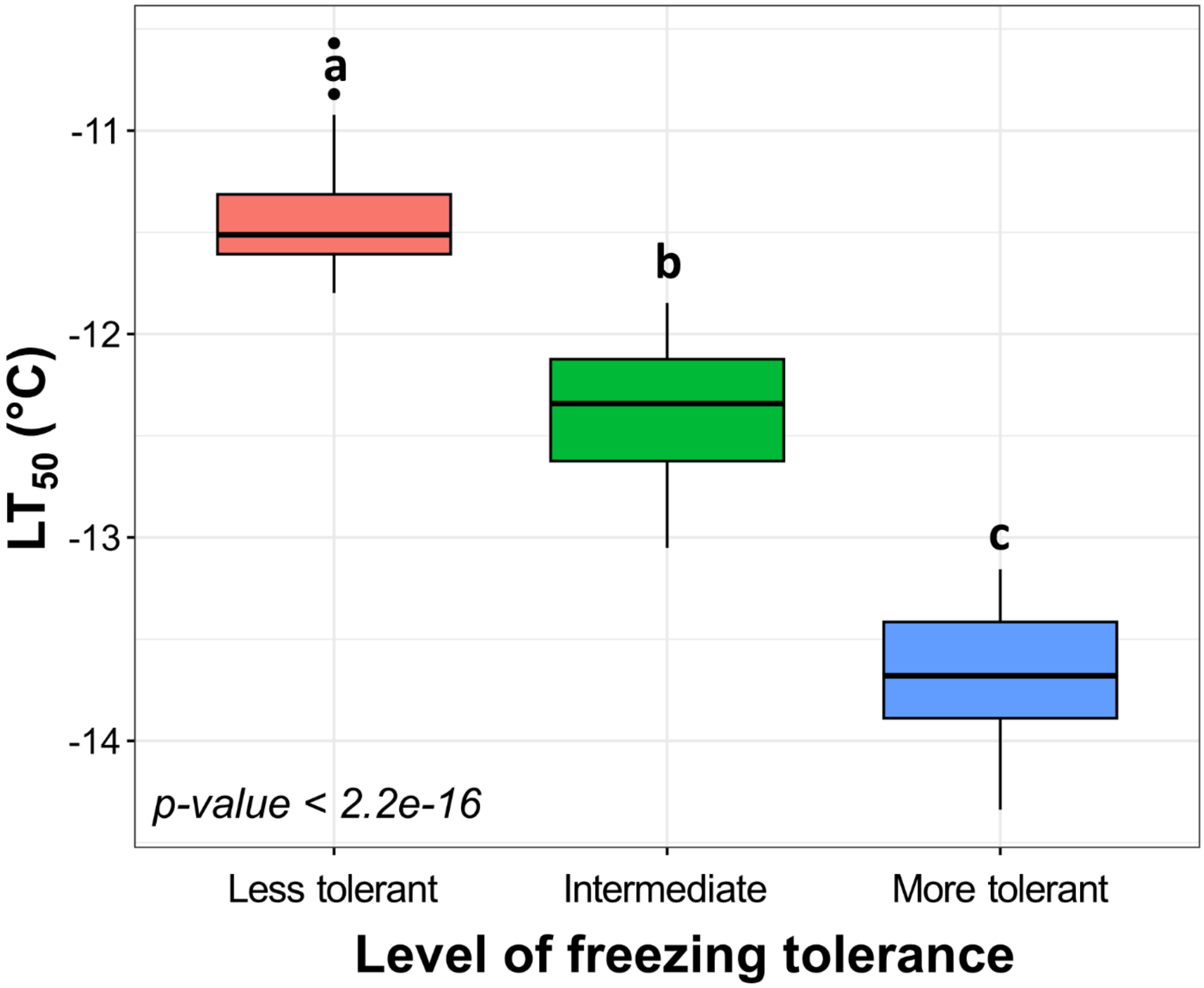

Figure S2. The 160 winter wheat genotypes were split into three groups of freezing tolerance according to their LT_{50} (temperature where 50% of plants are killed) values, obtained under controlled conditions. The less tolerant group ranges between -10.57 and -11.82 °C, the intermediate group ranges between -11.83 and -13.08 °C, and the more tolerant group ranges between -13.09 and -14.34 °C. The letters above the boxplot indicate significant ($p < 0.05$) differences between compared groups.

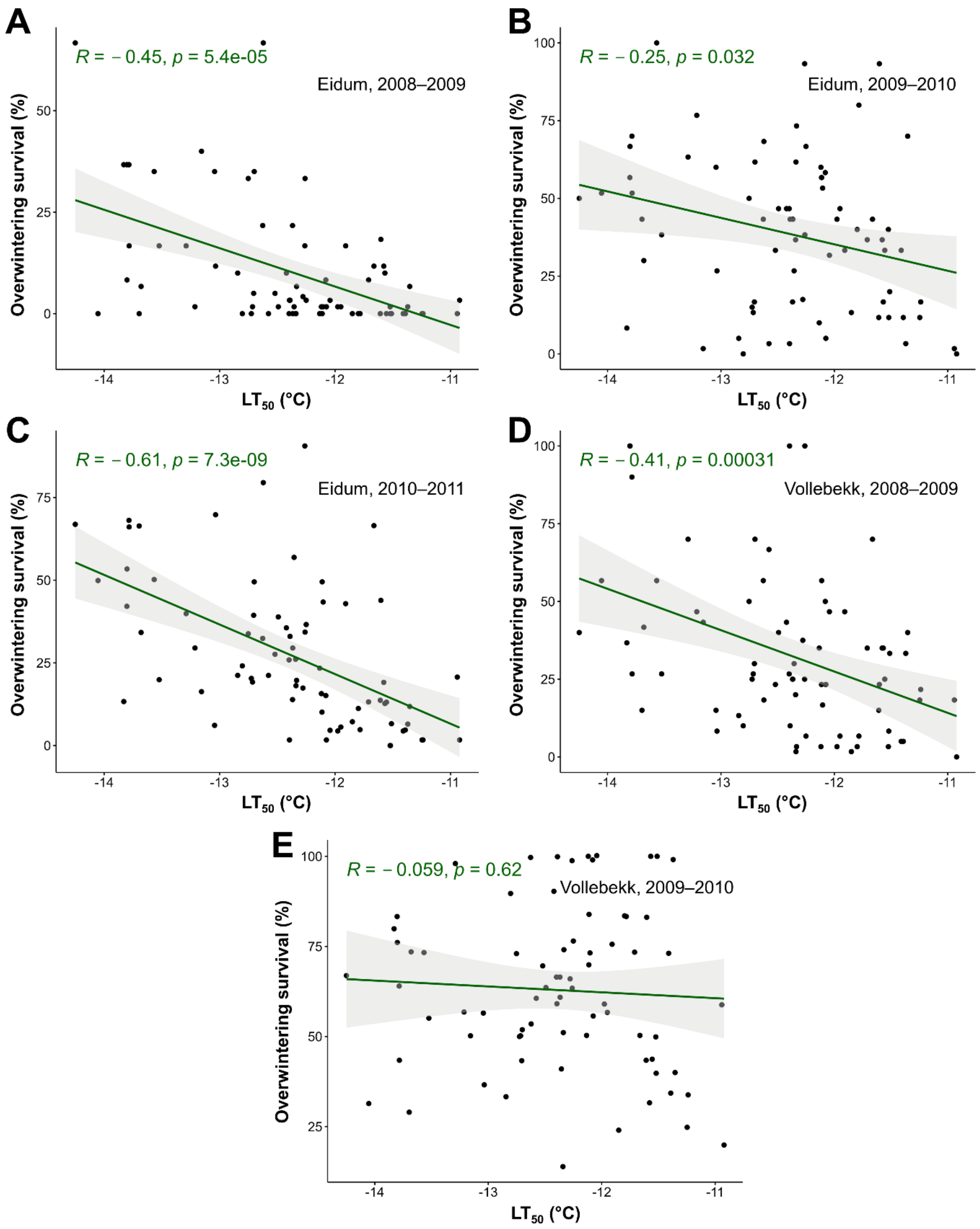

Figure S3. The relationship between mean LT50 (temperature where 50% of plants are killed) values and overwintering scores from field trials in Norway of 74 winter wheat cultivars and landraces of Nordic origin. The trials were carried out in Eidum, Stjørdal municipality during the winter seasons of 2008-2009 (**A**), 2009-2010 (**B**), 2010-2011 (**C**), and in Vollebekk, Ås municipality during the winter seasons of 2008-2009 (**D**) and 2009-2010 (**E**).

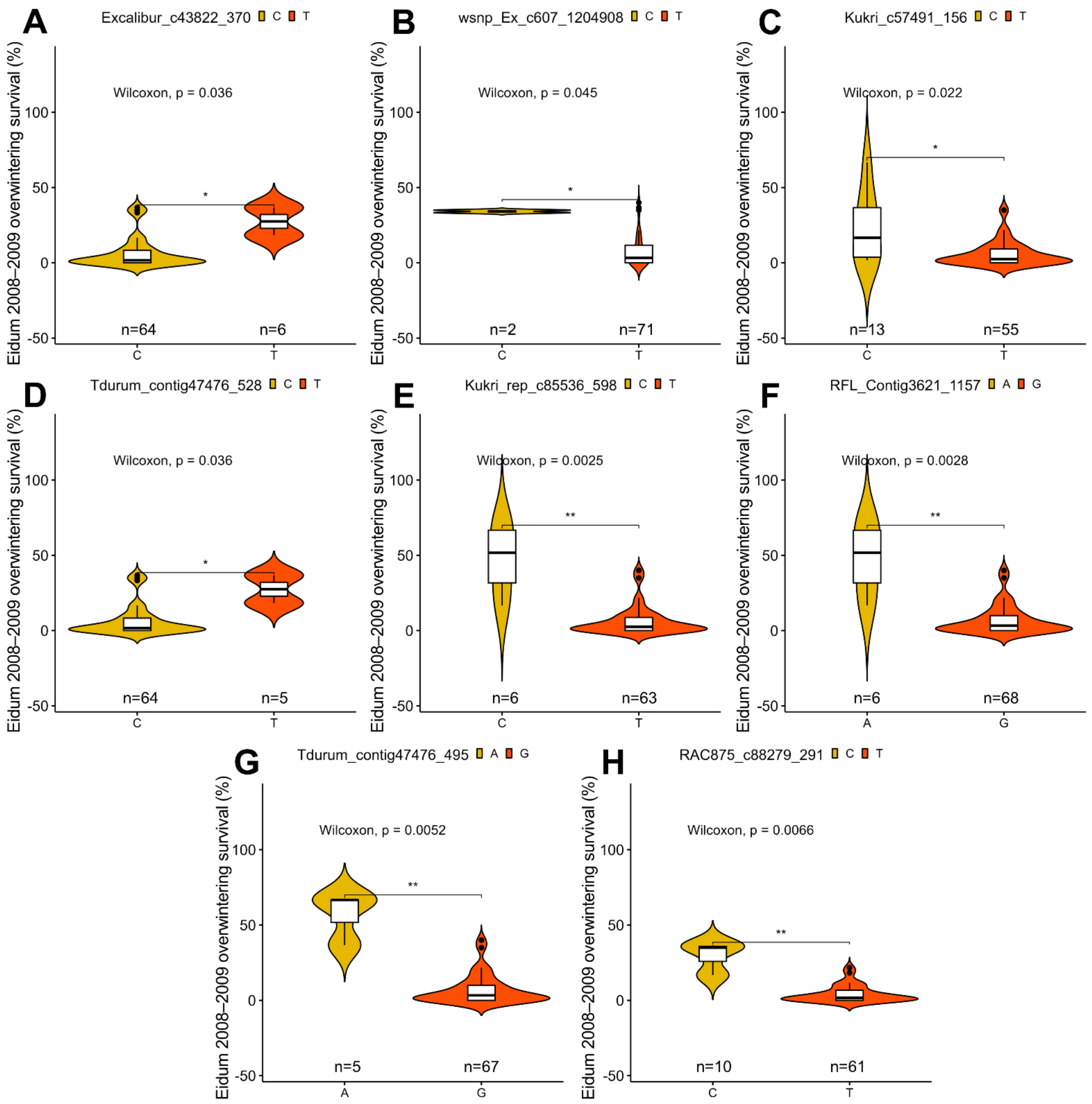

Figure S4. The effect of single nucleotide polymorphism (SNP) marker alleles on winter survival of wheat in the field. Winter survival was evaluated as the visual percentage of surviving plants after winter in the Eidum 2008-2009 field trial. Depicted are the markers with significant ($p < 0.05$) allele effect: Excalibur_c43822_370 (A), wsnp_Ex_c607_1204908 (B), Kukri_c57491_156 (C), Tdurum_contig47476_528 (D), Kukri_rep_c85536_598 (E), RFL_Contig3621_1157 (F), Tdurum_contig47476_495 (G), and RAC875_c88279_291 (H). * indicates significant differences at $p < 0.05$, ** - at $p < 0.01$. "n" refers to number of observations for each of the two major alleles within the population.

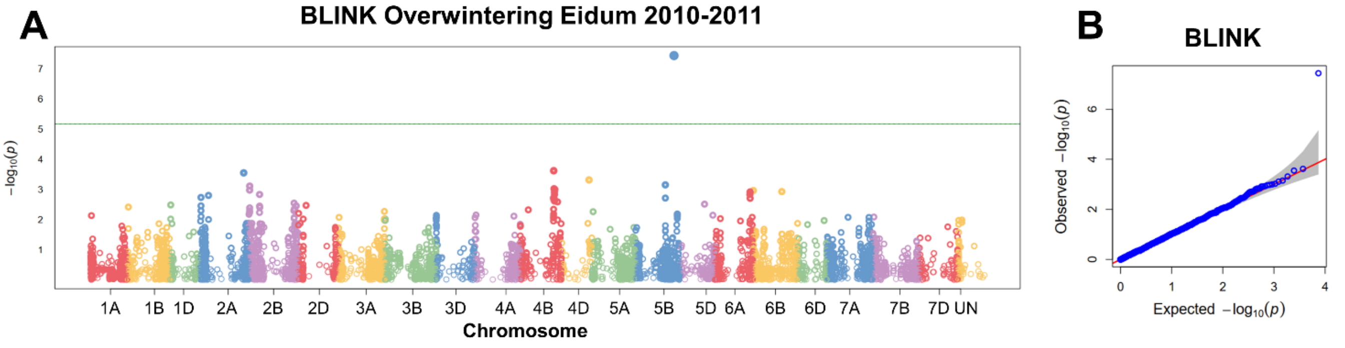

Figure S5. Manhattan plot of single nucleotide polymorphisms (SNPs), associated with winter survival, obtained using the GAPIT BLINK (**A**) model, and the corresponding quantile-quantile (Q-Q) plot (**B**). Winter survival was evaluated as the visual percentage of surviving plants after winter in the Eidum 2010-2011 field trial. The horizontal solid green line represents the Bonferroni cutoff, whereas the horizontal dashed green line represents the FDR cutoff. The gray area in the Q–Q plot indicates the 95% confidence interval under the null hypothesis that there is no association between the SNP and the investigated trait. Blue dots above the grey area represent the SNPs, associated with the investigated trait.

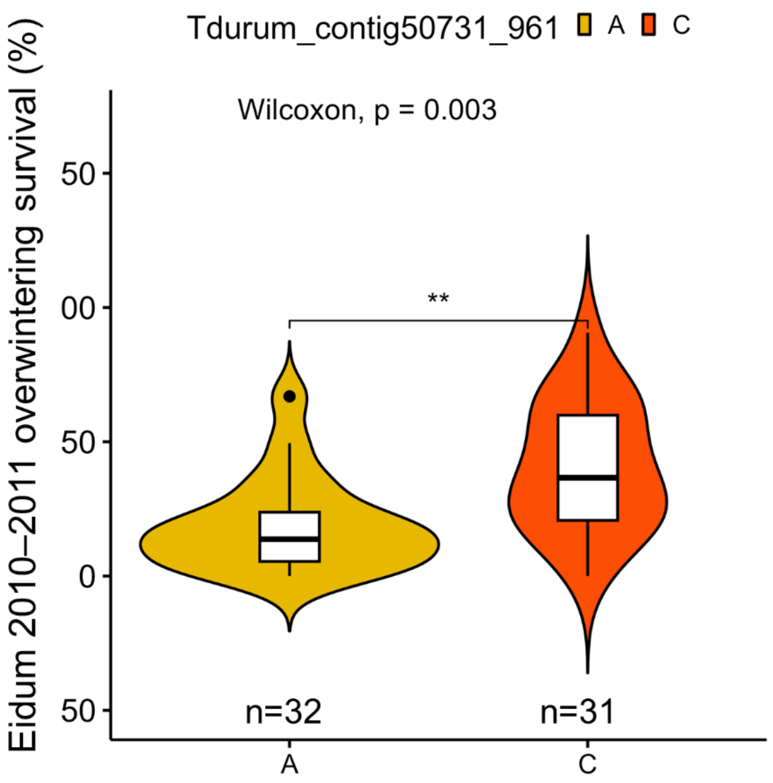

Figure S6. The effect of single nucleotide polymorphism (SNP) marker alleles on winter survival of wheat in the field. Winter survival was evaluated as the visual percentage of surviving plants after winter in the Eidum 2010-2011 field trial. Depicted is the marker with a significant ($p < 0.01$) allele effect (Tdurum_contig50731_961). "n" refers to number of observations for each of the two major alleles within the population.

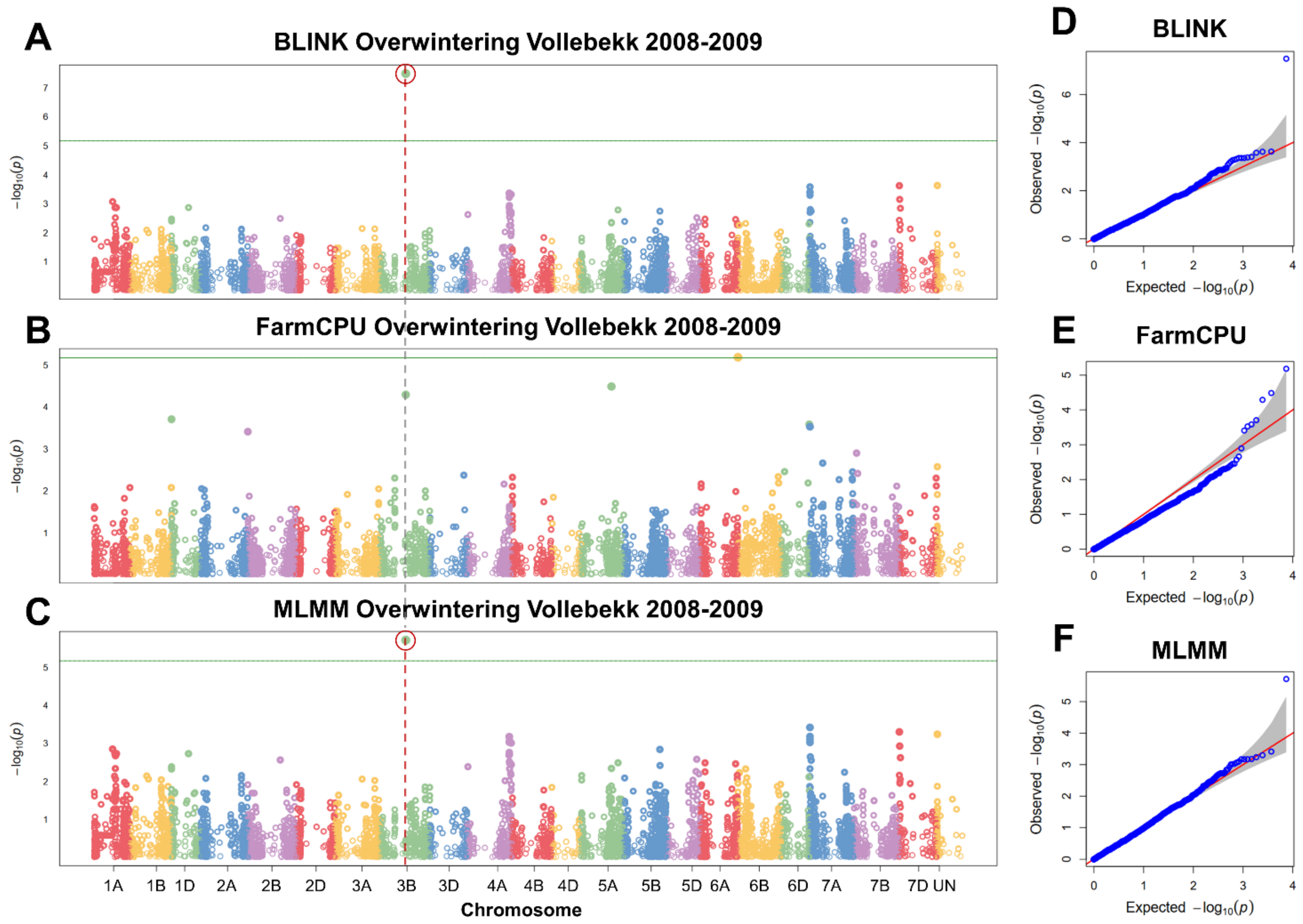

Figure S7. Manhattan plots of single nucleotide polymorphisms (SNPs), associated with winter survival, obtained using the GAPIT BLINK (**A**), FarmCPU (**B**) and MLMM (**C**) models, and corresponding quantile-quantile (Q-Q) plots (**D-F**). Winter survival was evaluated as the visual percentage of surviving plants after winter in the Vollebekk 2008-2009 field trial. The horizontal solid green line represents the Bonferroni cutoff, whereas the horizontal dashed green line represents the FDR cutoff. The SNPs significantly associated with winter survival according to multiple models are circled in red. The gray areas in the Q–Q plots indicate the 95% confidence interval under the null hypothesis that there is no association between the SNP and the investigated trait. Blue dots above the grey area represent the SNPs, associated with the investigated trait.

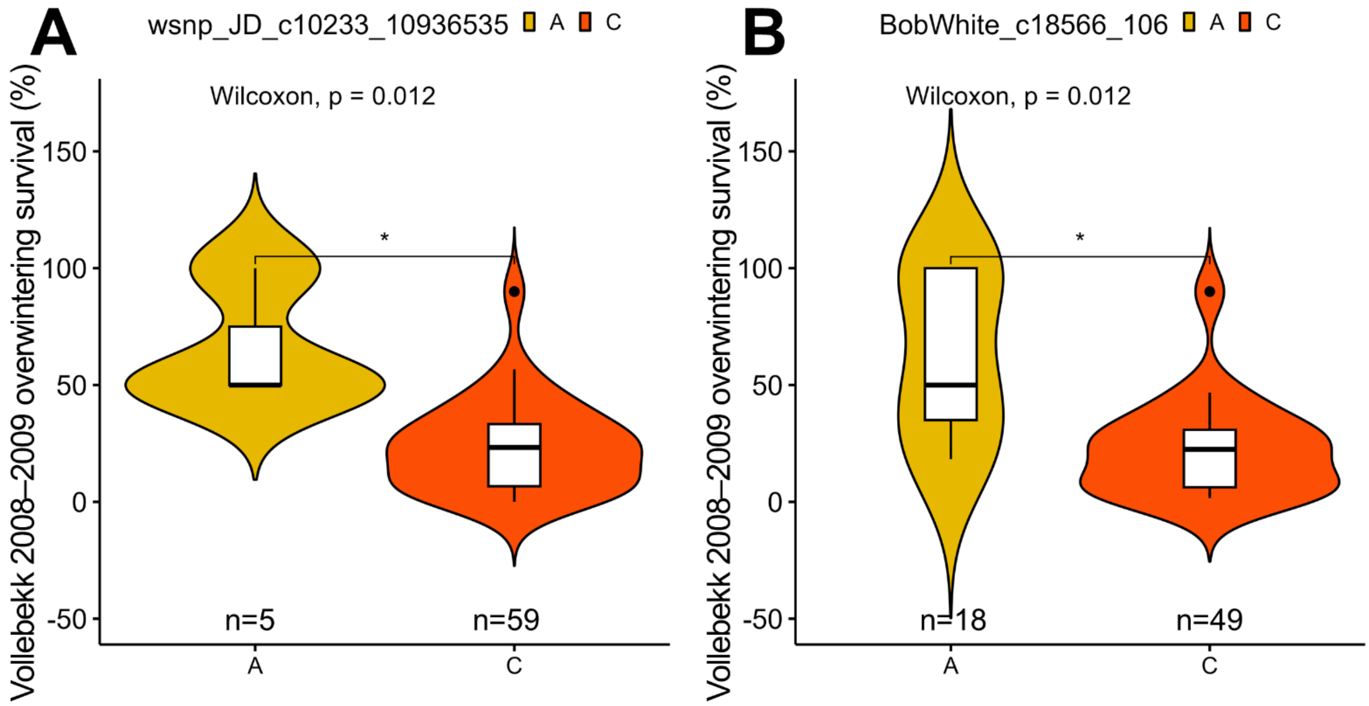

Figure S8. The effect of single nucleotide polymorphism (SNP) marker alleles on winter survival of wheat in the field. Winter survival was evaluated as the visual percentage of surviving plants after winter in the Vollebekk 2008-2009 field trial. Depicted are the markers with significant ($p < 0.05$) allele effect: wsnp_JD_c10233_10936535 (A), and BobWhite_c18566_106 (B). * indicates significant differences at $p < 0.05$. "n" refers to number of observations for each of the two major alleles within the population.

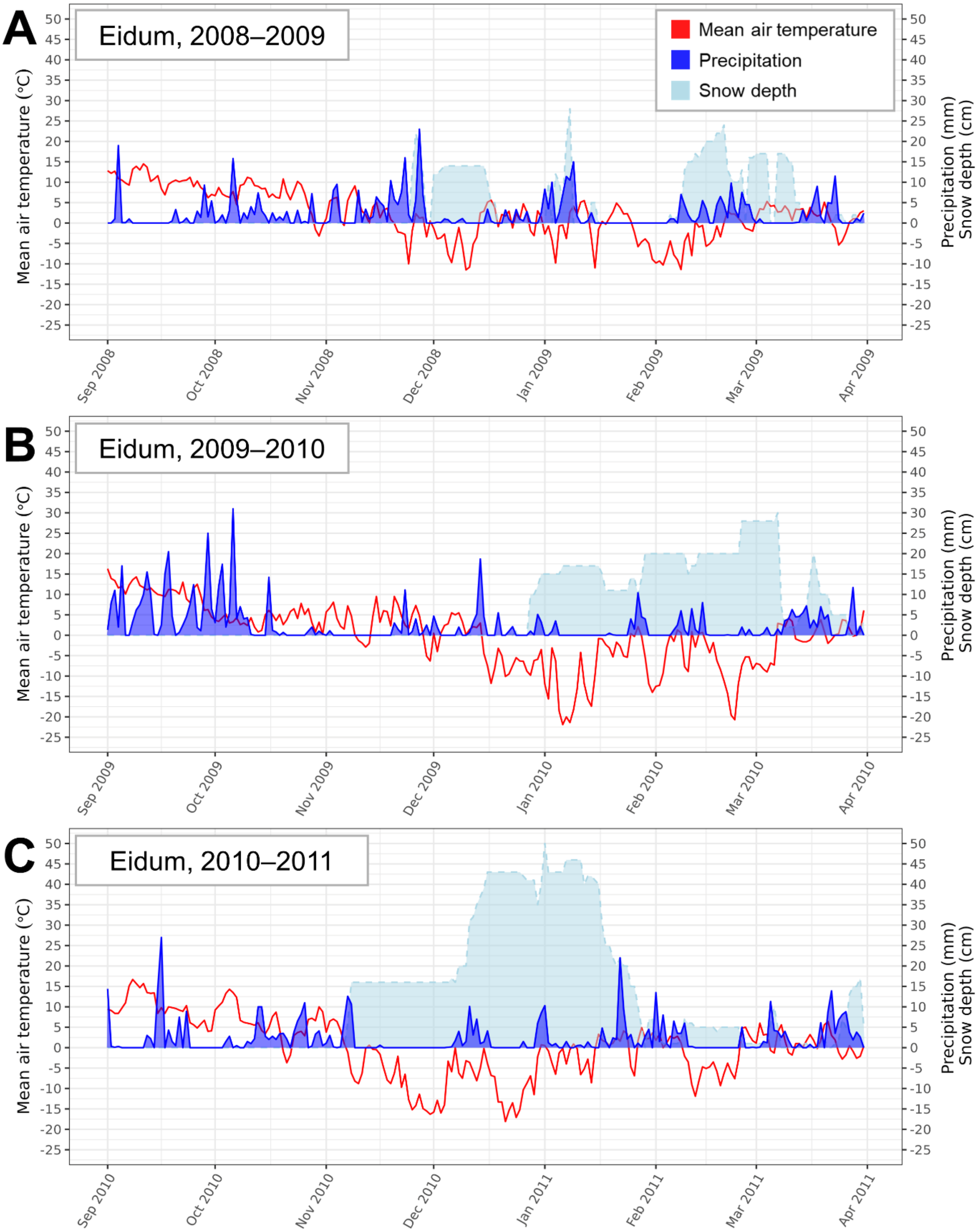

Figure S9. Meteorological conditions throughout the months of September – March in Eidum, Stjørdal municipality, Norway during the winter seasons of 2008-2009 (**A**), 2009-2010 (**B**) and 2010-2011 (**C**).

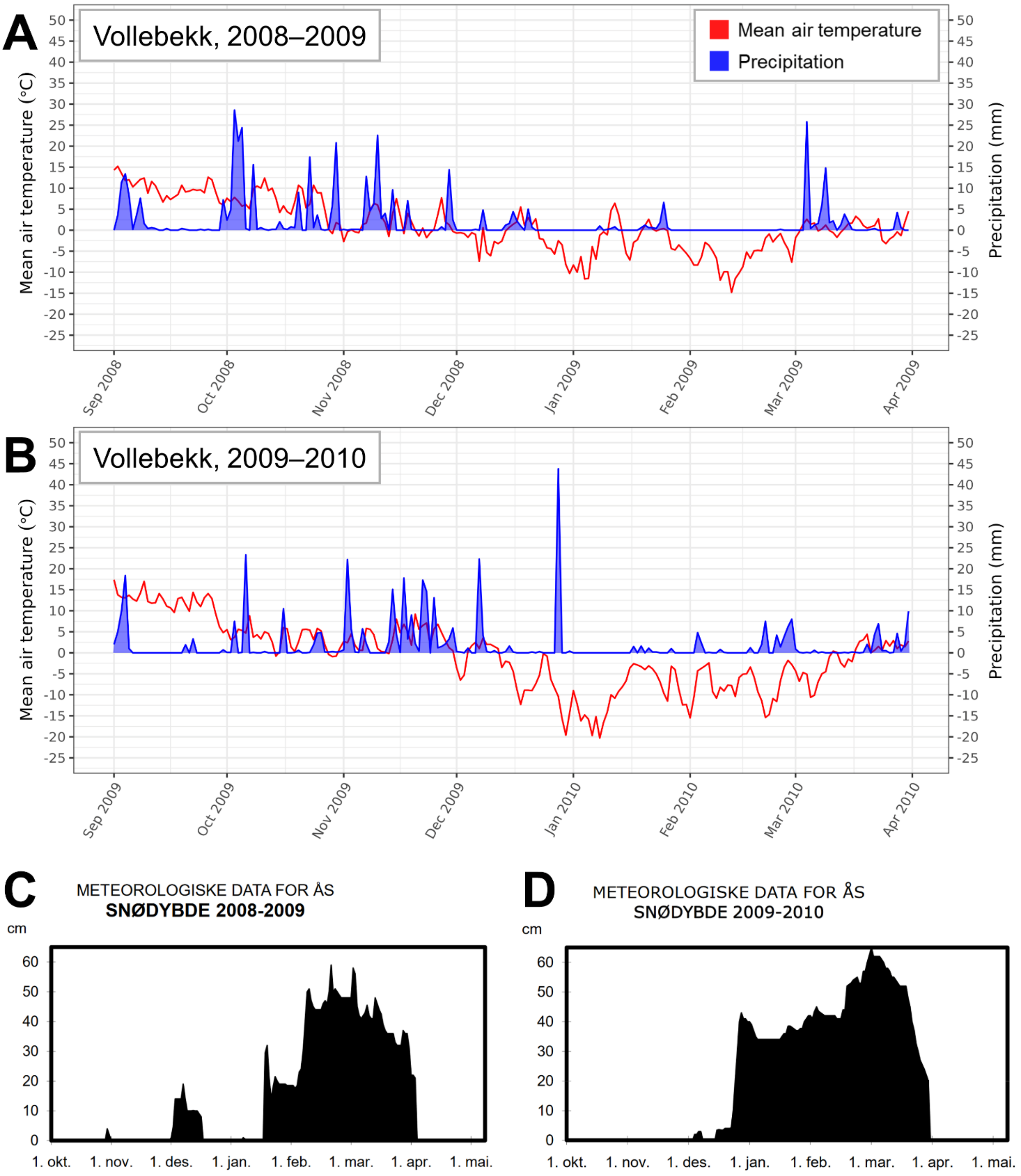

Figure S10. Meteorological conditions throughout the months of September – March in Vollebekk, Ås municipality, Norway during the winter seasons of 2008-2009 (**A**) and 2009-2010 (**B**). Snow depth data was not available in digital form and was obtained from meteorological reports for Ås by Hansen and Grimenes [1,2] (**C**,**D**).

References

1. Hansen, V.T.; Grimenes, A.A. *Meteorologiske Data for Ås 2009*; NMBU, Ås, Norway, 2010; ISBN 978-82-7636-023-3.
2. Hansen, V.T.; Grimenes, A.A. *Meteorologiske Data for Ås 2010*; NMBU, Ås, Norway, 2011; ISBN 978-82-7636-024-0.
